# canSAR 2024—an update to the public drug discovery knowledgebase

**DOI:** 10.1093/nar/gkae1050

**Published:** 2024-11-13

**Authors:** Phillip W Gingrich, Rezvan Chitsazi, Ansuman Biswas, Chunjie Jiang, Li Zhao, Joseph E Tym, Kevin M Brammer, Jun Li, Zhigang Shu, David S Maxwell, Jeffrey A Tacy, Ioan L Mica, Michael Darkoh, Patrizio di Micco, Kaitlyn P Russell, Paul Workman, Bissan Al-Lazikani

**Affiliations:** Department of Genomic Medicine; Therapeutics Discovery Division; and The Institute for Data Science in Oncology; University of Texas MD Anderson Cancer Center, Houston, TX 77030, USA; Department of Genomic Medicine; Therapeutics Discovery Division; and The Institute for Data Science in Oncology; University of Texas MD Anderson Cancer Center, Houston, TX 77030, USA; Department of Genomic Medicine; Therapeutics Discovery Division; and The Institute for Data Science in Oncology; University of Texas MD Anderson Cancer Center, Houston, TX 77030, USA; Department of Genomic Medicine; Therapeutics Discovery Division; and The Institute for Data Science in Oncology; University of Texas MD Anderson Cancer Center, Houston, TX 77030, USA; Department of Genomic Medicine; Therapeutics Discovery Division; and The Institute for Data Science in Oncology; University of Texas MD Anderson Cancer Center, Houston, TX 77030, USA; Enterprise Development and Integration, University of Texas MD Anderson Cancer Center, Houston, TX 77030, USA; Enterprise Development and Integration, University of Texas MD Anderson Cancer Center, Houston, TX 77030, USA; Enterprise Development and Integration, University of Texas MD Anderson Cancer Center, Houston, TX 77030, USA; Enterprise Development and Integration, University of Texas MD Anderson Cancer Center, Houston, TX 77030, USA; Department of Genomic Medicine; Therapeutics Discovery Division; and The Institute for Data Science in Oncology; University of Texas MD Anderson Cancer Center, Houston, TX 77030, USA; Enterprise Development and Integration, University of Texas MD Anderson Cancer Center, Houston, TX 77030, USA; Enterprise Development and Integration, University of Texas MD Anderson Cancer Center, Houston, TX 77030, USA; Department of Genomic Medicine; Therapeutics Discovery Division; and The Institute for Data Science in Oncology; University of Texas MD Anderson Cancer Center, Houston, TX 77030, USA; Department of Genomic Medicine; Therapeutics Discovery Division; and The Institute for Data Science in Oncology; University of Texas MD Anderson Cancer Center, Houston, TX 77030, USA; Department of Genomic Medicine; Therapeutics Discovery Division; and The Institute for Data Science in Oncology; University of Texas MD Anderson Cancer Center, Houston, TX 77030, USA; Centre for Cancer Drug Discovery, Division of Cancer Therapeutics, The Institute of Cancer Research, London SW7 3RP, UK; Department of Genomic Medicine; Therapeutics Discovery Division; and The Institute for Data Science in Oncology; University of Texas MD Anderson Cancer Center, Houston, TX 77030, USA

## Abstract

canSAR (https://cansar.ai) continues to serve as the largest publicly available platform for cancer-focused drug discovery and translational research. It integrates multidisciplinary data from disparate and otherwise siloed public data sources as well as data curated uniquely for canSAR. In addition, canSAR deploys a suite of curation and standardization tools together with AI algorithms to generate new knowledge from these integrated data to inform hypothesis generation. Here we report the latest updates to canSAR. As well as increasing available data, we provide enhancements to our algorithms to improve the offering to the user. Notably, our enhancements include a revised ligandability classifier leveraging Positive Unlabeled Learning that finds twice as many ligandable opportunities across the pocketome, and our revised chemical standardization pipeline and hierarchy better enables the aggregation of structurally related molecular records.

## Graphical Abstract

**Graphical Abstract:**
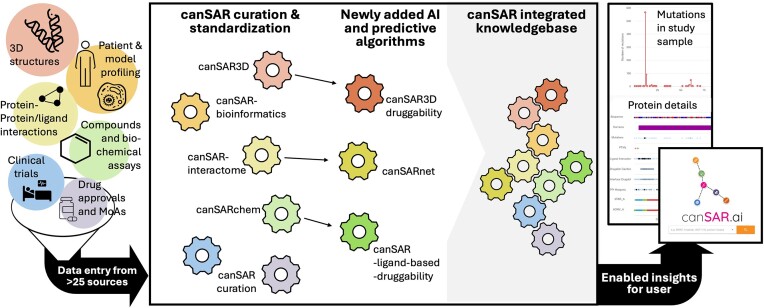

## Introduction

canSAR remains the largest public, integrative platform dedicated to cancer drug discovery (1–6). Following the initial success of the Human Genome Project (7), the accessibility and reliability of molecular profiling technologies have increased exponentially. These advances, combined with groundbreaking developments in structural biology, synthetic chemistry and computational modelling, have generated vast amounts of data that, if organized and linked appropriately, can deliver great insights to the drug discoverer.

However, when considered in isolation, these extensive datasets fall short of their potential impact on the drug discovery community. The true value of these resources is unlocked only through comprehensive integration, enabling a holistic understanding of the complex interplay between different biological, clinical, and chemical domains.

The original mission of canSAR was, and continues to be, to enable better data-driven decision-making and hypothesis generation in early drug discovery through the unification of otherwise siloed data and by applying machine learning predictors to address some of drug discovery's bottlenecks. Thus, users are empowered to gain rapid insights, and generate testable hypotheses supported by the relational information and annotations that canSAR provides.

Herein, we describe the latest data in canSAR—which are routinely updated from key resources such as ChEMBL (8–12), BindingDB (13,14), the Protein Data Bank (15–17), The Cancer Genome Atlas (TCGA) (18), and clinicaltrials.gov, to name a few. We further complement these data by filling key gaps through our own curation efforts and those from our collaborators such as the Chemical Probes Portal (19). We also continue to enhance our standardization tools and predictive algorithms that help generate insights. We highlight recent enhancements to our structural ligandability assessment, chemical standardization and registration processes, and overall usability. Finally, we illustrate through an example how the unique integrative capabilities can be used to generate testable hypotheses.

## Data at a glance

As of this writing, canSAR currently integrates data from across more than 25 data sources.

Whole Genome/Exome sequencing (WGS/WES) and RNA transcriptomic data are standardized and curated from key public resources including The Cancer Genome Atlas (18) and DepMap (20), among others. CanSAR currently contains WGS/WES data from 12 561 tumor samples relating to 12 520 patients and RNAseq data for 19 408 samples from 10 955 patients. In total, molecular profiling data come from across 19 thousand primary tumor samples and 1700 metastatic samples, with 4211 pediatric samples spanning both. In addition, canSAR contains molecular profiling data for 1921 cancer cell lines, as well as CRISPR knock out and drug sensitivity data for these cell lines.

Chemical and bioactivity data by compound and target are gathered from ChEMBL (8–12), BindingDB (13,14), and other sources, totaling >4.5 million compounds with over 13.3 million bioactivities, representing an increase of roughly 50% for both data types in canSAR since 2021 (5). Compounds and bioactivities can be linked to protein-specific structural information pulled from the Protein Data Bank and analyzed by our suite of pipelines. This results in over 200 thousand analyzed 3D protein structures with more than 6 million protein pockets and associated ligandability predictions for individual chains.

Protein-protein interactions are incorporated across 9 data sources (21–30). Through curation and standardization, we provide over 1 million interactions across 18 698 proteins. We additionally annotate confidence levels for these interactions based on the type and strength of experimental evidence (3).

Additionally, roughly 490 thousand clinical trials are incorporated into canSAR from clinicaltrials.gov. These data allow users to discover compounds investigated for specific cancers at a glance. Moreover, we have mapped these trials to our chemical and drug dictionaries as well as the Anatomical Therapeutic Chemical (ATC) classification (https://www.who.int/tools/atc-ddd-toolkit/atc-classification). This allows the user to search and filter clinical trials by compounds, therapeutic classes and more. Coupled with target mappings, bioactivity data, expression data, and mutation data across chemical and multi-omics data types, chemical starting points and repurposing opportunities can be investigated.

canSAR’s mission is to identify and integrate key data that help decision-making in early drug discovery and translational research. In cases where we identify key missing data, we have begun a systematic effort to source and curate these data by the canSAR team as well as our international collaborators. From drug synergies to target annotation to compound nomenclatures, our team endeavors to provide the most complete data resource possible to the community to enable and accelerate discovery efforts. Similarly, we apply state-of-the-art methodologies, whether they are developed by the canSAR team or others. We implemented new druggability assessment and chemical standardization methodologies to fill key methodological gaps currently not addressed by other publicly available methods as detailed in dedicated manuscripts describing these methods (31,32).

### 3D Ligandability predictions

canSAR applies a suite of machine learning algorithms to assess the suitability of any protein for therapeutic development across multiple modalities (small molecule ligandability, mAb accessibility, etc.). A key component of this evaluation is the 3D ligandability assessment. Here we define ligandability (sometimes referred to as druggability) as the availability of a cavity on the protein that has geometric and physicochemical properties consistent with the binding of a drug-like, synthetic molecule. To account for structural variations as much as possible for any given protein, we assess the ligandability across all available experimental structures of all proteins in the Protein Data Bank in Europe (PDBe) (15,17). We update these calculations weekly, ensuring the most up to date information to the drug discoverer. This enables researchers at one glance to evaluate therapeutic opportunities presented from all available structures including those from model organisms.

Our 3D ligandability assessment has undergone significant enhancements. Our previous method, like all other methods in the public domain, faced a strong inherent limitation. All such methods have been trained using standard supervised learning on curated datasets of protein structures bound to drugs or drug-like molecules. Pockets containing these compounds were taken as positives, and all other pockets were treated as negatives, even if those pockets were potentially ligandable or even bound in other structures. Labeling these pockets as negatives biases any model away from finding novel druggable sites in the standard supervised learning paradigm. Despite this limitation, our method was demonstrably successful at identifying novel druggable proteins that were later experimentally validated (2,33).

The notion of a negative ‘undruggable’ pocket is scientifically intangible since definitively labeling cavities as undruggable is impossible. Therefore, we redefined the inherent challenges of this task as a Positive Unlabeled (PU) Learning (34) problem, and we have transitioned to an iterative, PU Random Forest (RF) approach. This method allows us to refine our negative training set by confidently labeling most likely unligandable pockets, promoting enhanced recovery of potentially ligandable pockets by only learning ‘negative’ patterns from pockets strongly differentiated from ligandable pockets as confirmed by experiment. We have detailed this method elsewhere (31).

**Figure 1.**
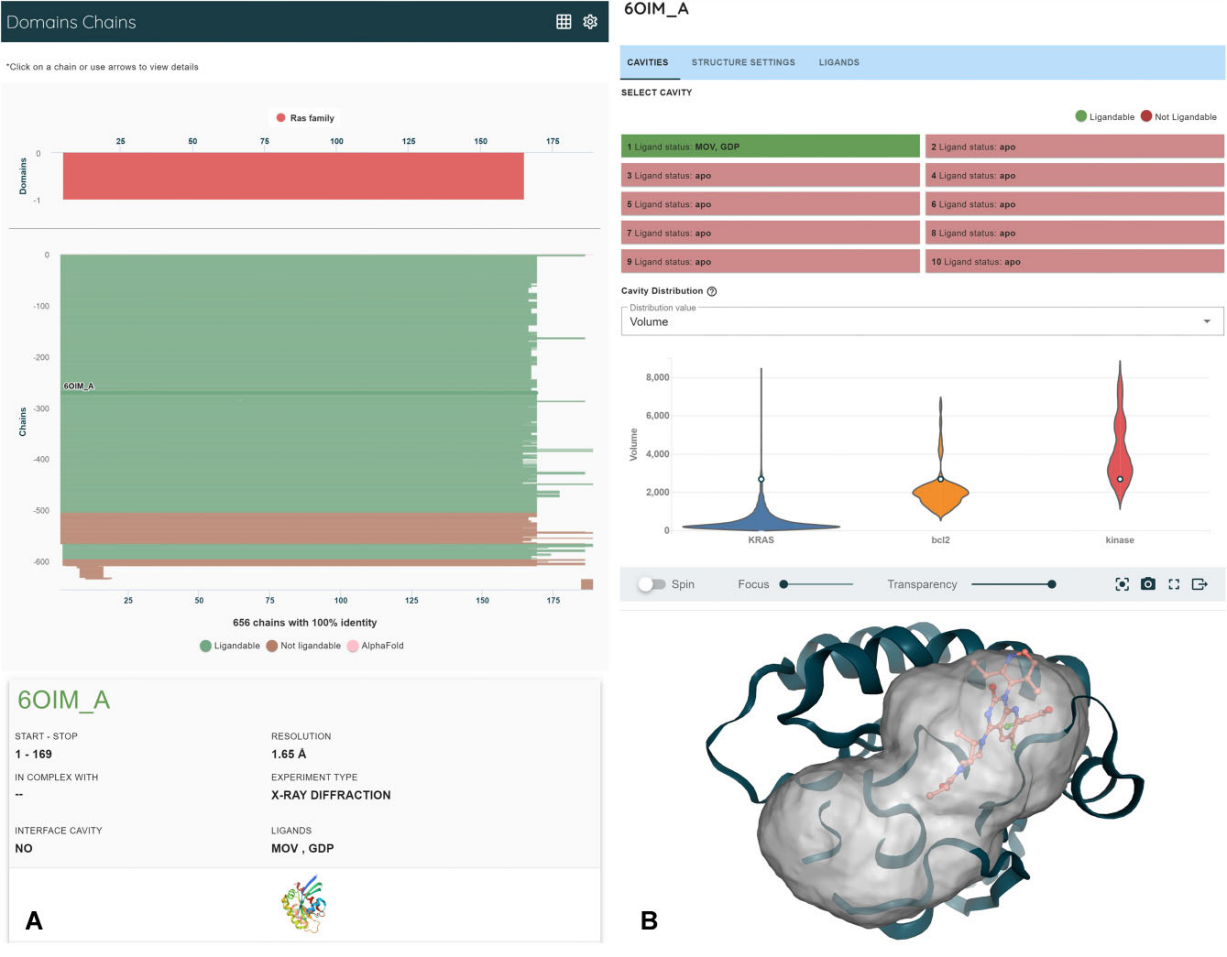
Structural ligandability for KRAS highlights accessibility to ligandable structures and pockets. (**A**) Color coding of ligandability and structural alignment by sequence and functional domains enables discovery of structures of interest and shows that there are 656 experimentally determined structural instances for KRAS, of which 535 are predicted to have a ligandable cavity (green). The top bar for every protein represents the AlphaFold2 model. (**B**) Each individual structure can be examined in detail. Ligandable pockets are visualized with present ligands and physicochemical property distributions.

Using this new PU algorithm, canSAR now contains over 6 million analyzed pockets from over 591 thousand experimentally solved protein chains derived from 204 thousand structures. To expand our predictions, we additionally assessed all pockets on human AlphaFold 2 (AF2) models (35). We remain restricted to AF2 because the AlphaFold 3 models and source code are unavailable in the public domain. From AF2, we identified another 200 thousands pockets. Our new PU-based predictive model has identified nearly 600 000 ligandable pockets across protein chains alone, with roughly 65% of those being unoccupied by a ligand, presenting potentially novel opportunities. This is an increase in the number of identified druggable pockets by more than two-fold compared to our previous model (approximately 250 000 pockets as ligandable with approximately 50% of those being unoccupied by a ligand). Additionally, 163 000 bioassemblies and 840 000 resulting interface cavities have been analyzed by our pipeline. Our updated classifier finds 24% of them as druggable, again doubling our previous druggable predictions and affording opportunities to disrupt protein-protein interactions. Figure 1 is a screenshot from the ligandability predictions for KRAS.

Aggregating to the protein level, we examined the number of human proteins found to contain a druggable pocket by the new canSAR classifier for both experimental structures and AlphaFold2 models. Across the PDB, we found that 77% of human proteins with experimentally determined structures contained at least one predicted druggable cavity. When considering all AlphaFold2 models of human proteins, we found 52% of the proteome to contain a druggable pocket. However, the confidences of AlphaFold2 models vary as measured by the average pLDDT score. We further reduced the list of druggable proteins to those with an average pLDDT score of >70 and found that only 42% of publicly available AlphaFold2 models contain a predicted druggable pocket *and* have high confidence structure on average. By considering both model confidence scores and druggability predictions, users may triage sites on predicted protein structures for investigation and leverage the growing body of predicted protein structures to find novel opportunities.

Given the PU nature of this problem, it is impossible to quantify the negative recall or the precision of our predictions. Despite our increases in positive predictions, the majority (91%) of pockets from individual protein chains are found to be undruggable, consistent with the general difficulty to identify targetable sites on proteins. The positively predicted sites represent opportunities for the community to pursue and validate experimentally.

While our revised classifier is deployed as of this writing, classification probabilities and associated confidence intervals are forthcoming in a minor release. This addition will provide users with additional means to assess pockets and proteins more subtly within their own expertise and unique contexts. In addition to our weekly structural analyses, we are committed to retraining and updating our models as available structural data continue to grow. canSAR’s pipelines are optimized for rapid calculation of druggability for experimentally-determined structures as well as computationally-predicted models. As soon as AlphaFold3 and other advanced predicted models become publicly available, they will be included alongside AlphaFold2 models in our analysis and druggability prediction pipelines.

### Chemistry and chemical hierarchies

The principal goal of integrating chemical data within canSAR is to map relevant portions of chemical space to bioactivity and structurally confirmed interactions for protein targets. This integration enables users not only to identify known active compounds or probes for specific targets but also to perform comprehensive chemical searches for related compounds, expanding the scope of chemical exploration within the platform.

**Figure 2.**
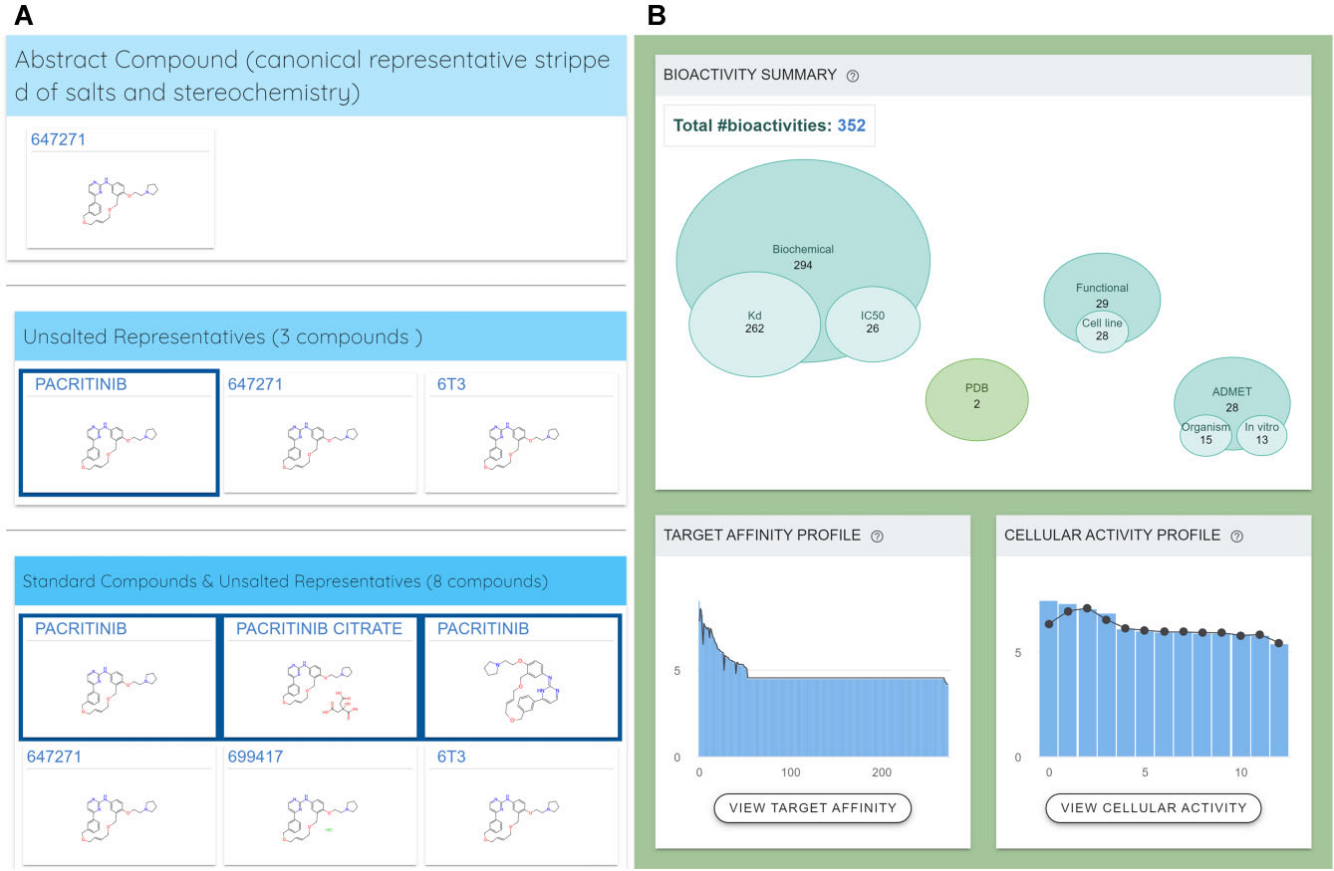
The chemical hierarchy for pacritinib illustrates how related structural forms of compounds as well as their associated bioactivities are aggregated. (**A**) Hierarchical ordering of molecules affords traceability of chemical records according to the performed chemical standardization transformations. (**B**) Bioactivities are integrated alongside associated chemical structural data, allowing for discovery of related records and activities.

We have previously demonstrated the value of extensive standardization of chemical structures including tautomers in the development of canSARchem (36). That enabled us to bring together key data for bioactive small molecules that had been artificially separated in different databases due to lack of sophisticated standardization. However, our prior pipeline required the use of commercial software and was computationally intensive, limiting the ambition of data growth in canSAR. Therefore, we revised our chemical hierarchy and standardization pipeline to automatically and rapidly address these challenges, which we describe in detail elsewhere (32). Briefly, our updated standardization pipeline ensures that all chemical entries are uniformly processed by (a) assessing input for physical consistency, (b) removing irrelevant fragments, solvents and counter-ions, (c) tautomerically canonicalizing compounds, and d) flattening stereochemistry and isotopic labels. This meticulous standardization allows compounds and their associated bioactivities to be integrated according to a hierarchical generalization that supports more effective querying and analysis. As an example, the different tautomeric forms of pacritinib in CHEMBL2035187 and CHEMBL5095049 previously failed to correctly merge to the same canonical tautomer. Figure 2 highlights (A) the correct hierarchical organization of related structures of pacritinib and (B) the resulting aggregation of associated bioactivities.

In addition to fingerprint and substructure searching, canSAR incorporates Bemis-Murcko scaffolds (37) to allow users to identify structurally related compounds with greater ease and precision. This scaffold-based approach affords the discovery of related bioactivity information across structurally related molecules to serve as starting points for experimental testing.

**Figure 3.**
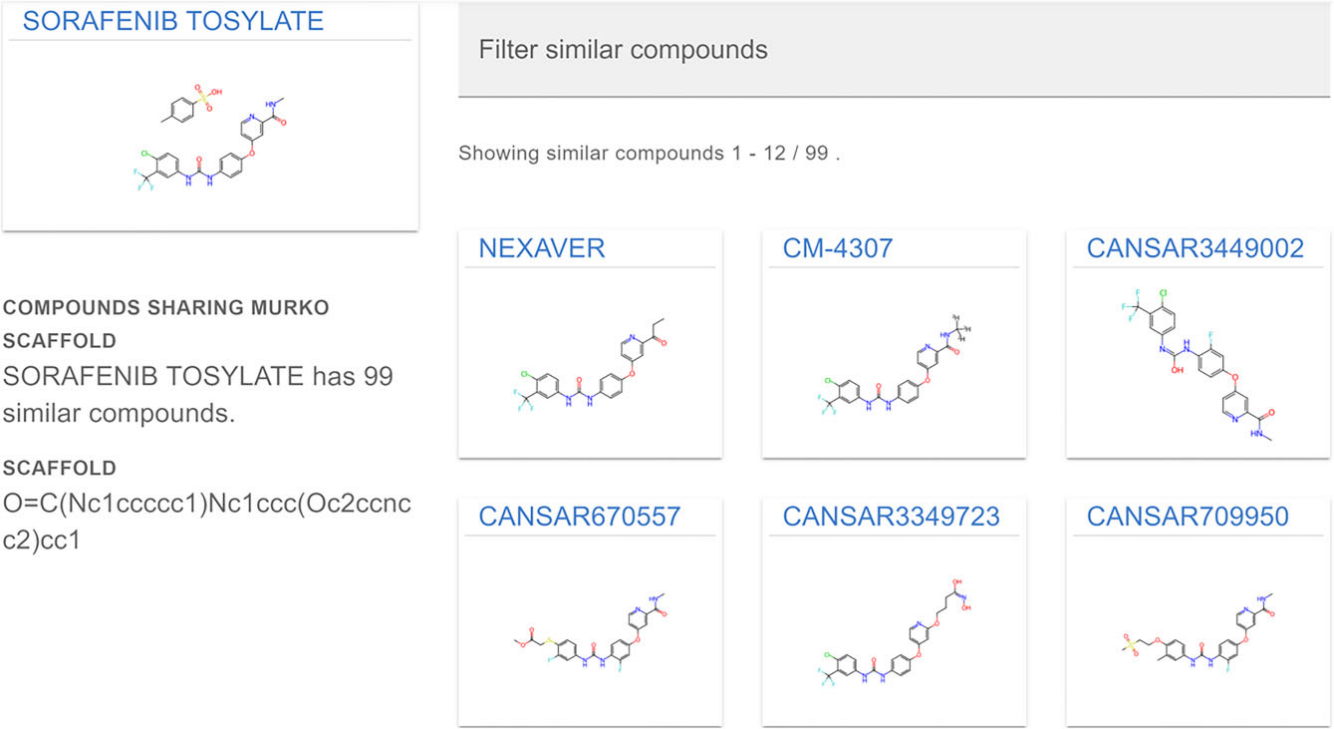
Scaffold searching for sorafenib tosylate. Fragments and isotopic labeling are now correctly removed from Bemis-Murcko Scaffolds. This allows for improved discovery of structurally related compounds and, by extension, associated bioactivities.

Previously, scaffolds were generated for each molecule in the chemical hierarchy with the retention of isotopic labels and solvent or counter-ion fragments. This could result in missing otherwise related molecular records during scaffold searches. For instance, sorafenib, ^11^C-sorafenib, and sorafenib tosylate were previously not grouped by their molecular scaffold. Our revised pipeline now ensures that all variants of sorafenib share the correct scaffold, eliminating these discrepancies. Figure 3 highlights this revision for sorafenib tosylate, where CM-4307, a deuterated variant, is now correctly found by scaffold searching.

Lastly, our revised pipeline has been fully implemented completely in python, leveraging only open-source software. In summary, these changes have increased the number of compounds within canSAR to roughly 4.5 million compounds. As we continue to grow the available chemical data within canSAR, we aim to utilize our improved scalability to completely automate the integration of new chemical data from our sources, keeping canSAR updated for the community.

### Integrative tools enable hypothesis generation—an example with KRAS

**Figure 4.**
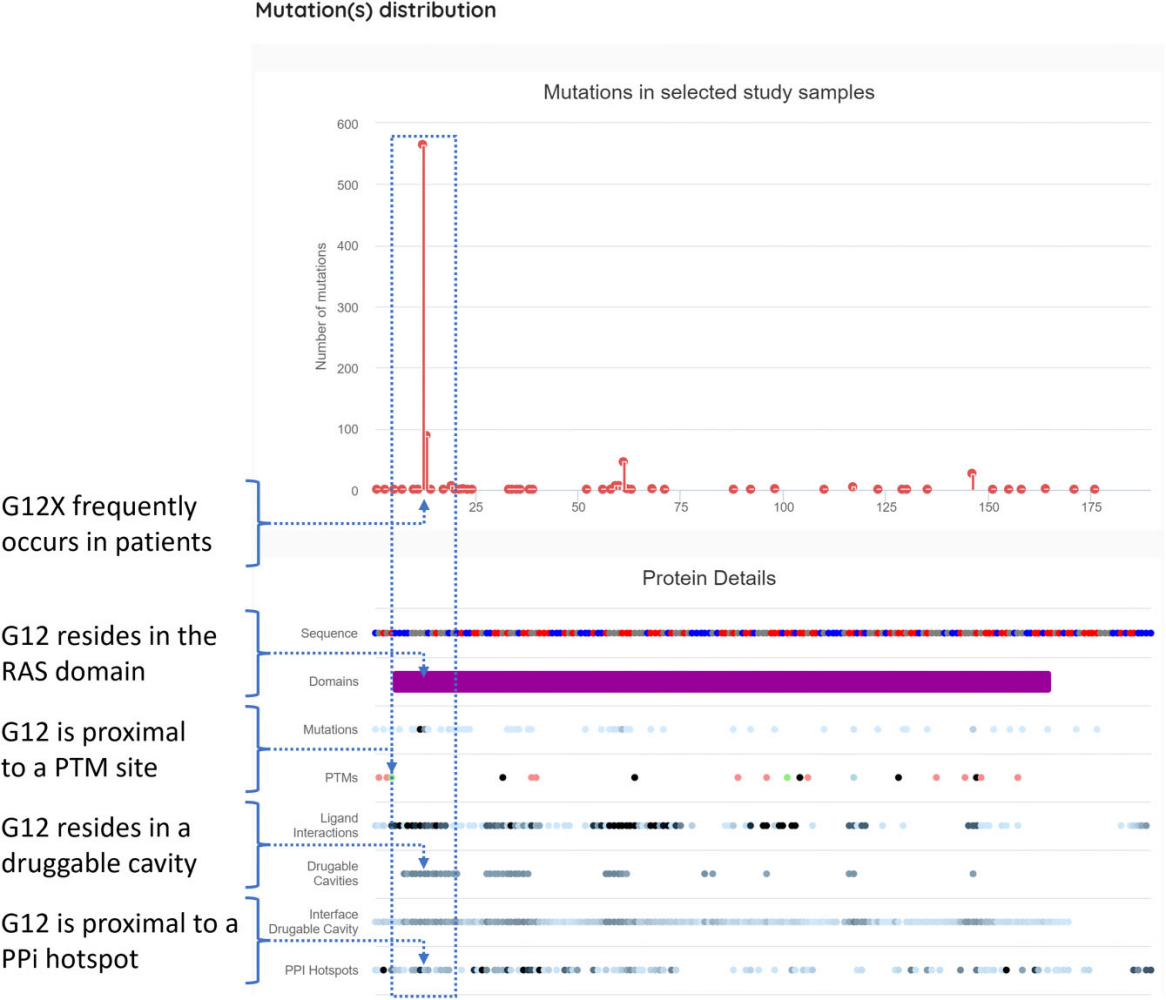
The ‘Mutations’ page for KRAS. This visualization integrates mutational frequency and types, functional annotations, protein–protein interaction sites and ligandability predictions, allowing users to identify opportunities in sequence space with likely functional consequence.

The power of canSAR is in the integration of data across genomic, structural, and chemical spaces. By combining, analyzing, and visualizing both original data and predictions from our models, otherwise inaccessible insights are readily available. Below we demonstrate an example—a researcher identifies a mutation on a gene of interest and wishes to explore its potential for the discovery of a novel therapeutic. Figure 4 presents a ‘vertical’ integration of functional, ligandability, and mutation data for KRAS through this fully interactive canSAR view. Beginning with mutation data, the high occurrence of G12X mutations is shown in the lollipop plot. The lanes beneath represent different functional annotations for each amino acid in the protein. The integration immediately reveals that this mutation is proximal in sequence space to post-translational modification (PTM) sites and is present in multiple discovered druggable cavities. Additionally, a fraction of these druggable cavities exists at protein-protein interaction sites. This analysis would suggest there is a druggable site encompassing amino acid G12, which is likely to play an important function due to its proximity to a PTM and its involvement in protein interactions. Users can utilize this visualization to rapidly pinpoint opportunities on proteins of interest. In the case of KRAS, this is consistent with community knowledge of its therapeutic targeting (38). This type of visual analysis democratizes these data for non-data scientists, allowing the broader cancer drug discovery community to find opportunities to meet clinical needs.

### Usability improvements

In the latest release of canSAR, enhancements have been made to improve usability and data accessibility. We have introduced advanced download functionalities. Additionally, we have implemented several ‘under the hood’ upgrades that significantly enhance the overall user experience. These upgrades collectively enhance the usability, efficiency, and accessibility of canSAR, aligning with our ongoing commitment to support the research community with cutting-edge tools and resources.

New download features have been integrated in various sections of canSAR. Notably, users can now download CSV files containing Approved and Investigational Drugs directly from the Synopsis overview of any Molecular Target page. Additionally, structure availability tables are also available for download. These data enable data mining and offline analyses, and we will continue to enable data availability from canSAR.

## Conclusion

canSAR continues its mission to democratize knowledge for early drug discovery and translational research efforts to the cancer community. It does so through integrating complex multidisciplinary data across multi-omics, structural biology, systems biology, pharmacology, and medicinal chemistry; and providing insights that can only be gleaned through full integration. Moreover, canSAR layers predictive AI algorithms on these data to help guide the identification of novel druggable opportunities. The enhancements detailed in this update—available at https://cansar.ai—bring significant improvements to machine learning predictions, chemical aggregation methods and bioinformatics analyses, further empowering the research community.

Looking ahead, our team and our international collaborators remain committed to the ongoing growth and evolution of canSAR. Future updates will focus on incorporating deep-learning approaches for more advanced ligandability assessments and enhanced chemical property and activity predictions. We will continue our horizon scanning to ensure that we implement state-of-the-art methodologies whether they are developed by the canSAR team or others, ensuring that canSAR remains a powerful tool for drug discovery.

## Data Availability

canSAR is freely available at https://cansar.ai.

## References

[1] Halling-Brown M.D. , Bulusu K.C., Patel M., Tym J.E., Al-Lazikani B. canSAR: an integrated cancer public translational research and drug discovery resource. Nucleic Acids Res. 2012; 40:D947–D956. 22013161 10.1093/nar/gkr881 PMC3245005

[2] Bulusu K.C. , Tym J.E., Coker E.A., Schierz A.C., Al-Lazikani B. canSAR: updated cancer research and drug discovery knowledgebase. Nucleic Acids Res. 2014; 42:D1040–D1047. 24304894 10.1093/nar/gkt1182 PMC3964944

[3] Tym J.E. , Mitsopoulos C., Coker E.A., Razaz P., Schierz A.C., Antolin A.A., Al-Lazikani B. canSAR: an updated cancer research and drug discovery knowledgebase. Nucleic Acids Res. 2016; 44:D938–D943. 26673713 10.1093/nar/gkv1030 PMC4702774

[4] Coker E.A. , Mitsopoulos C., Tym J.E., Komianou A., Kannas C., Di Micco P., Villasclaras Fernandez E., Ozer B., Antolin A.A., Workman P. canSAR: update to the cancer translational research and drug discovery knowledgebase. Nucleic Acids Res. 2019; 47:D917–D922. 30496479 10.1093/nar/gky1129 PMC6323893

[5] Mitsopoulos C. , Di Micco P., Fernandez E.V., Dolciami D., Holt E., Mica I.L., Coker E.A., Tym J.E., Campbell J., Che K.H. canSAR: update to the cancer translational research and drug discovery knowledgebase. Nucleic Acids Res. 2021; 49:D1074–D1082. 33219674 10.1093/nar/gkaa1059 PMC7778970

[6] Di Micco P. , Antolin A.A., Mitsopoulos C., Villasclaras-Fernandez E., Sanfelice D., Dolciami D., Ramagiri P., Mica I.L., Tym J.E., Gingrich P.W. canSAR: update to the cancer translational research and drug discovery knowledgebase. Nucleic Acids Res. 2023; 51:D1212–D1219. 36624665 10.1093/nar/gkac1004 PMC9825411

[7] Lander E.S. , Linton L.M., Birren B., Nusbaum C., Zody M.C., Baldwin J., Devon K., Dewar K., Doyle M., FitzHugh W. et al. Initial sequencing and analysis of the human genome. Nature. 2001; 409:860–921. 11237011 10.1038/35057062

[8] Gaulton A. , Bellis L.J., Bento A.P., Chambers J., Davies M., Hersey A., Light Y., McGlinchey S., Michalovich D., Al-Lazikani B. ChEMBL: a large-scale bioactivity database for drug discovery. Nucleic Acids Res. 2012; 40:D1100–D1107. 21948594 10.1093/nar/gkr777 PMC3245175

[9] Bento A.P. , Gaulton A., Hersey A., Bellis L.J., Chambers J., Davies M., Krüger F.A., Light Y., Mak L., McGlinchey S. The ChEMBL bioactivity database: an update. Nucleic Acids Res. 2014; 42:D1083–D1090. 24214965 10.1093/nar/gkt1031 PMC3965067

[10] Gaulton A. , Hersey A., Nowotka M., Bento A.P., Chambers J., Mendez D., Mutowo P., Atkinson F., Bellis L.J., Cibrián-Uhalte E. The ChEMBL database in 2017. Nucleic Acids Res. 2017; 45:D945–D954. 27899562 10.1093/nar/gkw1074 PMC5210557

[11] Mendez D. , Gaulton A., Bento A.P., Chambers J., De Veij M., Félix E., Magariños M.P., Mosquera J.F., Mutowo P., Nowotka M. et al. ChEMBL: towards direct deposition of bioassay data. Nucleic Acids Res. 2019; 47:D930–D940. 30398643 10.1093/nar/gky1075 PMC6323927

[12] Zdrazil B. , Felix E., Hunter F., Manners E.J., Blackshaw J., Corbett S., de Veij M., Ioannidis H., Lopez D.M., Mosquera J.F. The ChEMBL Database in 2023: a drug discovery platform spanning multiple bioactivity data types and time periods. Nucleic Acids Res. 2024; 52:D1180–D1192. 37933841 10.1093/nar/gkad1004 PMC10767899

[13] Liu T. , Lin Y., Wen X., Jorissen R.N., Gilson M.K. BindingDB: a web-accessible database of experimentally determined protein–ligand binding affinities. Nucleic Acids Res. 2007; 35:D198–D201. 17145705 10.1093/nar/gkl999 PMC1751547

[14] Gilson M.K. , Liu T., Baitaluk M., Nicola G., Hwang L., Chong J. BindingDB in 2015: a public database for medicinal chemistry, computational chemistry and systems pharmacology. Nucleic Acids Res. 2016; 44:D1045–D1053. 26481362 10.1093/nar/gkv1072 PMC4702793

[15] Velankar S. , Best C., Beuth B., Boutselakis C., Cobley N., Sousa Da Silva A., Dimitropoulos D., Golovin A., Hirshberg M., John M. PDBe: protein data bank in Europe. Nucleic Acids Res. 2010; 38:D308–D317. 19858099 10.1093/nar/gkp916 PMC2808887

[16] Burley S.K. , Berman H.M., Kleywegt G.J., Markley J.L., Nakamura H., Velankar S. Protein Data Bank (PDB): the single global macromolecular structure archive. Protein Crystallography: Methods and Protocols. 2017; 627–641. 10.1007/978-1-4939-7000-1_26 PMC5823500 28573592

[17] Armstrong D.R. , Berrisford J.M., Conroy M.J., Gutmanas A., Anyango S., Choudhary P., Clark A.R., Dana J.M., Deshpande M., Dunlop R. PDBe: improved findability of macromolecular structure data in the PDB. Nucleic Acids Res. 2020; 48:D335–D343. 31691821 10.1093/nar/gkz990 PMC7145656

[18] Weinstein J.N. , Collisson E.A., Mills G.B., Shaw K.R., Ozenberger B.A., Ellrott K., Shmulevich I., Sander C., Stuart J.M. The cancer genome atlas pan-cancer analysis project. Nat. Genet. 2013; 45:1113–1120. 24071849 10.1038/ng.2764 PMC3919969

[19] Antolin A.A. , Sanfelice D., Crisp A., Villasclaras Fernandez E., Mica I.L., Chen Y., Collins I., Edwards A., Müller S., Al-Lazikani B. et al. The Chemical Probes Portal: an expert review-based public resource to empower chemical probe assessment, selection and use. Nucleic Acids Res. 2023; 51:D1492–D1502. 36268860 10.1093/nar/gkac909 PMC9825478

[20] Tsherniak A. , Vazquez F., Montgomery P.G., Weir B.A., Kryukov G., Cowley G.S., Gill S., Harrington W.F., Pantel S., Krill-Burger J.M. Defining a cancer dependency map. Cell. 2017; 170:564–576. 28753430 10.1016/j.cell.2017.06.010 PMC5667678

[21] Oughtred R. , Rust J., Chang C., Breitkreutz B.J., Stark C., Willems A., Boucher L., Leung G., Kolas N., Zhang F. The BioGRID database: a comprehensive biomedical resource of curated protein, genetic, and chemical interactions. Protein Sci. 2021; 30:187–200. 33070389 10.1002/pro.3978 PMC7737760

[22] Damle N.P. , Köhn M. The human DEPhOsphorylation database DEPOD: 2019 update. Database. 2019; 2019:baz133. 31836896 10.1093/database/baz133 PMC6911163

[23] Luck K. , Kim D.-K., Lambourne L., Spirohn K., Begg B.E., Bian W., Brignall R., Cafarelli T., Campos-Laborie F.J., Charloteaux B. A reference map of the human binary protein interactome. Nature. 2020; 580:402–408. 32296183 10.1038/s41586-020-2188-x PMC7169983

[24] Breuer K. , Foroushani A.K., Laird M.R., Chen C., Sribnaia A., Lo R., Winsor G.L., Hancock R.E., Brinkman F.S., Lynn D.J. InnateDB: systems biology of innate immunity and beyond—Recent updates and continuing curation. Nucleic Acids Res. 2013; 41:D1228–D1233. 23180781 10.1093/nar/gks1147 PMC3531080

[25] Del Toro N. , Shrivastava A., Ragueneau E., Meldal B., Combe C., Barrera E., Perfetto L., How K., Ratan P., Shirodkar G. The IntAct database: efficient access to fine-grained molecular interaction data. Nucleic Acids Res. 2022; 50:D648–D653. 34761267 10.1093/nar/gkab1006 PMC8728211

[26] Hornbeck P.V. , Zhang B., Murray B., Kornhauser J.M., Latham V., Skrzypek E. PhosphoSitePlus, 2014: mutations, PTMs and recalibrations. Nucleic Acids Res. 2015; 43:D512–D520. 25514926 10.1093/nar/gku1267 PMC4383998

[27] Milacic M. , Beavers D., Conley P., Gong C., Gillespie M., Griss J., Haw R., Jassal B., Matthews L., May B. The reactome pathway knowledgebase 2024. Nucleic Acids Res. 2024; 52:D672–D678. 37941124 10.1093/nar/gkad1025 PMC10767911

[28] Lo Surdo P. , Iannuccelli M., Contino S., Castagnoli L., Licata L., Cesareni G., Perfetto L. SIGNOR 3.0, the SIGnaling network open resource 3.0: 2022 update. Nucleic Acids Res. 2023; 51:D631–D637. 36243968 10.1093/nar/gkac883 PMC9825604

[29] Essaghir A. , Demoulin J.-B. A minimal connected network of transcription factors regulated in human tumors and its application to the quest for universal cancer biomarkers. PLoS One. 2012; 7:e39666. 22761861 10.1371/journal.pone.0039666 PMC3382591

[30] Han H. , Cho J.-W., Lee S., Yun A., Kim H., Bae D., Yang S., Kim C.Y., Lee M., Kim E. TRRUST v2: an expanded reference database of human and mouse transcriptional regulatory interactions. Nucleic Acids Res. 2018; 46:D380–D386. 29087512 10.1093/nar/gkx1013 PMC5753191

[31] Gingrich P. , Biswas A., Di Micco P., Russell K.P., Al-Lazikani B. Positiveunlabelled learning applied to experimental structures and alpha-fold models expands the druggable proteome. 2024; ChemRxiv doi:16 September 2024, pre-print: not peer-reviewed 10.26434/chemrxiv-2024-mn4wm.

[32] Chitsazi R. , Gingrich P.W., Long J.P., Wu C., Al-Lazikani B. OpencanSARchem: chemistry registration and standardization pipeline for FAIR integration. 2024; ChemRxiv doi:16 September 2024, pre-print: not peer-reviewed 10.26434/chemrxiv-2024-mn4wm.

[33] Patel M.N. , Halling-Brown M.D., Tym J.E., Workman P., Al-Lazikani B. Objective assessment of cancer genes for drug discovery. Nat. Rev. Drug Discov. 2013; 12:35–50. 23274470 10.1038/nrd3913

[34] Bekker J. , Davis J. Learning from positive and unlabeled data: a survey. Mach. Learn. 2020; 109:719–760.

[35] Jumper J. , Evans R., Pritzel A., Green T., Figurnov M., Ronneberger O., Tunyasuvunakool K., Bates R., Žídek A., Potapenko A. Highly accurate protein structure prediction with AlphaFold. Nature. 2021; 596:583–589. 34265844 10.1038/s41586-021-03819-2 PMC8371605

[36] Dolciami D. , Villasclaras-Fernandez E., Kannas C., Meniconi M., Al-Lazikani B., Antolin A.A. canSAR chemistry registration and standardization pipeline. J. Cheminformatics. 2022; 14:28. 10.1186/s13321-022-00606-7 PMC9148294 35643512

[37] Bemis G.W. , Murcko M.A. The properties of known drugs. 1. Molecular frameworks. J. Med. Chem. 1996; 39:2887–2893. 8709122 10.1021/jm9602928

[38] Huang L. , Guo Z., Wang F., Fu L. KRAS mutation: from undruggable to druggable in cancer. Signal Transduct. Target. Ther. 2021; 6:386. 34776511 10.1038/s41392-021-00780-4 PMC8591115

